# Prevalence and Correlates of Clinically Elevated Depressive Symptoms in a Nationwide Sample of Transgender, Nonbinary, and Gender Diverse Young Adults in the United States: Cross-Sectional Survey Study

**DOI:** 10.2196/66630

**Published:** 2025-03-24

**Authors:** Sari Reisner, Yuxin Liu, Regina Tham, Kaiden Kane, S Wilson Cole, Elizabeth R Boskey, Sabra L Katz-Wise, Alex S Keuroghlian, Rena Xu

**Affiliations:** 1 Department of Epidemiology, University of Michigan School of Public Health, SPH-I 2649A, 1415 Washington Heights, Ann Arbor, MI, 48109, United States, 1 571-243-7532; 2 Department of Epidemiology, Harvard T.H. Chan School of Public Health, Boston, MA, United States; 3 The Fenway Institute, Fenway Health, Boston, MA, United States; 4 Department of Urology, Boston Children's Hospital, Boston, MA, United States; 5 Query Research Consulting, Glen Burnie, MD, United States; 6 Division of Gynecology, Boston Children’s Hospital, Boston, MA, United States; 7 Department of Surgery, Harvard Medical School, Boston, MA, United States; 8 Division of Adolescent/Young Adult Medicine, Boston Children's Hospital, Boston, MA, United States; 9 Department of Pediatrics, Harvard Medical School, Boston, MA, United States; 10 Department of Social and Behavioral Sciences, Harvard T.H. Chan School of Public Health, Boston, MA, United States; 11 Department of Psychiatry, Massachusetts General Hospital, Boston, MA, United States; 12 Psychiatry, Harvard Medical School, Boston, MA, United States

**Keywords:** transgender, depression, preventive screening, young adult, LGBTQ+, nonbinary, gender minority, gender diverse, mental health, prevalence, cross-sectional, survey, questionnaire, nationwide, USA, United States, North America

## Abstract

**Background:**

In the United States, transgender, nonbinary, and gender diverse (TGD) young adults experience a higher risk of depression compared to their cisgender peers. Understanding factors associated with increased risk of depression within the TGD young adult population is important to guide clinical care as well as inform the development of interventions to reduce mental health disparities.

**Objective:**

This exploratory study investigated the prevalence and correlates of positive screening for depressive symptoms among TGD young adults to inform the design, development, and implementation of national interventions aimed at improving mental health in this at-risk population.

**Methods:**

In August 2022, a cross-sectional, nationwide online survey was conducted among TGD young adults aged 18‐25 (N=104) in the United States. Measures included sociodemographic variables, family characteristics, mental health care utilization, and the two-item Patient Health Questionnaire-2 (PHQ-2) screener for depression. Poisson regression models with robust variance estimation were fitted to estimate adjusted prevalence ratios (aPR) and 95% CI for correlates of PHQ-2 depression (score ≥3).

**Results:**

The study sample had a mean age of 22 (SD 2) years; 48/104 (46%) individuals identified as Black, Indigenous, or other People of Color, and 69/104 (66%) were nonbinary. Overall, 44 (42%) individuals screened positive for depression using PHQ-2. In a multivariable model adjusted for age, race and ethnicity, US census region, and health insurance status, factors associated with increased depression prevalence using PHQ-2 included low versus high family support (aPR 1.54, 95% CI 1.05‐2.27) and identifying with a nonChristian religion versus being unaffiliated (aPR 1.66, 95% CI 1.04‐2.63). Factors associated with reduced depression prevalence included living in a rural versus suburban area (aPR 0.48, 95% CI 0.26‐0.92) and receiving mental health therapy versus not (aPR 0.71, 95% CI 0.53‐0.97).

**Conclusions:**

The high prevalence of depressive symptoms among TGD young adults in this study sample highlights the need for comprehensive mental health evaluation and support in this population. Depression risk is increased among certain subgroups, such as those with low family support. These findings are valuable in informing the development of interventions that aim to improve mental health outcomes among TGD young people.

## Introduction

In the United States, major depression is a leading cause of morbidity and mortality, affecting an estimated 21 million adults aged ≥18 years (8.3% of all US adults) [1,2]. It is most prevalent among young adults aged 18‐25 (18.6%) [3]. The peak incidence of depression occurs between ages 12‐25 years, and 1 in 5 adolescents (20.1%) aged 12‐17 in the US has had at least one major depressive episode, highlighting the importance of early detection and intervention efforts [3,4]. The US Preventive Services Task Force recommends routine preventive screening for depression among the general adult population aged ≥18 years in primary care settings, using tools such as the Patient Health Questionnaire (PHQ) to identify individuals who should be further evaluated for depression and facilitate appropriate referrals to mental health services [5-7].

Transgender, nonbinary, and gender diverse (TGD) young adults in the United States have a two- to four-fold higher risk of major depression [8] and are more likely to screen positive for clinically elevated depressive symptoms relative to their cisgender counterparts [9]. For example, in a national random sample of 65,231 college students drawn from 71 US college campuses, the prevalence of past 2-week depression using PHQ-9 was 57.8% for TGD young adults compared to 28.4% for cisgender young adults [9]. Other studies report similarly high prevalence rates of past 2-week elevated depressive symptoms, which vary depending on the screening instrument used and specific age range sampled: 56.7% (PHQ-9 depression in a clinical TGD sample aged 13‐20 y) [10], 57.9% (two-item Patient Health Questionnaire, PHQ-2 depression in a representative school-based sample of TGD 9th and 11th graders in Minnesota) [11], and 75% (PHQ-2 depression in nonprobability online sample of 5753 TGD young people aged 13‐24 y) [12]. Additional research is needed to understand factors associated with screening positive for depression among TGD young people, particularly using brief instruments appropriate for universal screening in clinical settings. Identifying TGD young people at increased risk for depression may facilitate more effective clinical care and inform the development of interventions to address mental health disparities.

Sociodemographic differences in depression prevalence based on gender identity and race or ethnicity have been inconsistent across studies among TGD young people [13], although nonbinary individuals reported consistently high rates of depression [9]. Other sociodemographic factors, such as geographic region and urbanity, are also important to consider, particularly in the context of legislation restricting elements of care for TGD populations. Several studies document the adverse negative health impacts of antitransgender legislation, even among TGD people who do not live in states with active or pending legislation [14]. While family support is protective for TGD mental health [15,16], other family-related factors, such as religiosity, necessitate further exploration [11], particularly since TGD young people have reported mixed quantitative findings on religiosity and mental health [17], and attitudes toward TGD people may differ by religious affiliation [18,19]. Additionally, both current utilization of mental health services and a history of seeking resources or information about mental health services may be relevant for future intervention implementation for TGD young adults. Consistent with a transformative paradigm in mental health that emphasizes early prevention and intervention of emerging mental health disparities in individuals aged 12‐25 years [20], patterns of adolescent help-seeking may persist into young adulthood. Some TGD young people may have sought mental health information, while others may not have, highlighting the need to better understand whether help-seeking behaviors differ between those with and without depression. This knowledge can inform targeted intervention strategies to reach those who may need help.

This exploratory study sought to investigate the prevalence and correlates of depression using PHQ-2 in a nationwide US sample of TGD young adults to understand factors associated with increased risk of depression among TGD young people and to inform the development of interventions aimed at reducing mental health disparities in this population.

## Methods

### Setting

In August 2022, a cross-sectional survey was conducted with a nationwide US sample of young adults recruited and enrolled through Prolific, a survey platform that hosts a diverse panel of adults aged ≥18 years. The survey was conducted as part of TransHealthGUIDE, a National Institutes of Health (NIH)-funded project (U01MH136558) aimed at designing and implementing strategies to address mental health disparities among TGD young adults. Early in-depth key informant interviews with TGD young adults, caregivers, and health care providers identified the need to better understand the context of depression among TGD young adults. Specifically, the interviews highlighted the need for further information on the characteristics of TGD young adults with and without depression, the role of family, mental health service utilization, peer support, and recalled help-seeking behaviors in adolescence. This exploratory self-reported survey was developed as an extension of the key informant interviews and was designed to expand upon and quantify each of these factors for intervention development.

### Participants

Eligibility criteria included: age 18‐25 years, TGD identity (self-identifying as TGD or having a gender identity different from sex assigned at birth), and US residency. The sample was stratified to enroll approximately 50 Black, Indigenous, and People of Color (BIPOC) people and 50 White young people. A total of 104 individuals were surveyed; additional details on survey methodology can be found elsewhere [21].

### Assessments

#### PHQ-2 Depression Status

The psychometrically validated PHQ-2 assessed the frequency of depressed mood and anhedonia over the past two weeks [22]. The two items were (1) “little interest or pleasure in doing things” and (2) “feeling down, depressed, or hopeless.” The response options were 0=not at all, 1=several days, 2=more than half the days, 3=nearly every day. The two items were highly correlated (*r*=0.81; *P*<.001) and summed. Scores ranged from 0‐6, and a score ≥3 was considered a positive screen for depression, based on a previously validated threshold for screening for major depression (sensitivity: 83%, specificity: 92%) [22].

#### Sociodemographic Variables, Family Context, Mental Health Services, Peer Support, and Mental Health Help-Seeking in Adolescence

Sociodemographic variables included age in years (continuous and categorized: 18‐20, 21‐23, 23‐25), race or ethnicity (ie, BIPOC monoracial, BIPOC multiracial, White), gender identity (ie, transgender men, transgender women, nonbinary), US Census region (ie, Northeast, Midwest, South, West), geographic context (ie, urban, suburban, rural), health insurance status (ie, private, public, both, none), and self-identified religion (eg, Agnostic, Atheist, Baptist, Buddhist, Catholic, Christian, Evangelical Protestant, Hindu, Jehovah’s Witness, Jewish, Latter-day Saint or Mormon, Mainline Protestant, Muslim, Orthodox Christian, Pagan, Sikh, Spiritual but not religious, Unitarian/Universalist, Unaffiliated, Other, not applicable; coded as Christian, not Christian, unaffiliated, more than one religion, or not applicable).

Family-related variables included family support, assessed using the question: “How much trouble did your parents or caregivers have accepting your gender identity?” Responses were coded as high family support (none/a little), low family support (a moderate amount/a lot), or not applicable (not applicable/they don’t know I’m trans); and family religion was self-reported by the young adult (using the same categories as for self-identified religion and coded as Christian, not Christian, unaffiliated, more than one religion, or not applicable).

Participants were queried on mental health services and peer support, including receiving care from a mental health therapist (yes, no) and whether they had ever participated in a peer support group (yes, no). Recalled frequency of mental health help-seeking in adolescence was assessed using 0-to-4-point Likert scales: (1) “When you were a teenager, how often were you looking for information about finding mental health support or a therapist?” (0=never to 4=very often; categorized as never/occasionally versus sometimes/often/very often), (2) “How useful would it have been to have reliable information on finding mental health support or a therapist?” (0=not at all to 4=extremely; categorized as not at all/slightly vs moderately/very/extremely), and (3) “How interested were you in getting therapy related to your gender? (0=not at all to 4=extremely; categorized as not at all/slightly, moderately/very/extremely, or not applicable).

### Study Size

This exploratory survey was designed to be complementary to key informant interviews conducted with TGD young adults, caregivers, and health care providers as part of the NIH-funded TransHealthGUIDE project. After an initial round of key informant interviews, the research team identified the need for feedback from a larger group of diverse TGD young adults, than those were accessible for the interviews, and designed this survey to capture additional feedback.

### Data Analysis

Descriptive statistics (ie, frequencies, percentages, means, standard deviations) summarized variables for the overall sample and by PHQ-2 depression status. Bivariate tests (2-tailed *t* tests for continuous variables; *χ*^*2*^ tests for binary or categorical variables) were used to compare participants with and without depression detected using PHQ-2. Poisson regression models with robust variance were fit to estimate prevalence ratios (PR) and 95% CI for correlates of depression [23]. Bivariate models were fit for each variable, followed by a single multivariable model with all variables to estimate adjusted prevalence ratios (aPR). Variables for modeling were selected *a priori* through a review of existing TGD research, conceptual salience, and findings from key informant interviews. Analyses were conducted using R software (version 4.4.3; R Foundation for Statistical Computing) [24] with statistical significance at *P*<.05 [24].

### Ethical Considerations

After review, the study protocol was deemed exempt by the Boston Children’s Hospital Institutional Review Board (IRB-P00043127). This study was performed in accordance with the ethical standards outlined in the 1964 Declaration of Helsinki and its subsequent amendments. Participants completed an informed consent process. This was an anonymized survey, and participants indicated consent by opting into the survey. Participants were compensated $3 for completing the survey in accordance with the standards of the Prolific survey platform.

## Results

### Study Sample

Sample characteristics are displayed in Table 1 for the total sample and stratified by PHQ-2 depression scores. Overall, 44 of 104 (42%) of the sample had clinically elevated depressive symptoms.

**Table 1.** Characteristics of a US nationwide sample of Transgender, Nonbinary, and Gender Diverse young adults aged 18‐25 years, stratified by Patient Health Questionnaire-2 (PHQ-2)^a^ depression^b^ .

Variables	Participants (N=104)	Depression^b^ (n=44), n (%)	No depression^b^ (n=60), n (%)	Bivariate test: *t* test (*df*) or *χ*^2^ test (*df*)^c^	*P* value
Age (years), mean (SD)	21.8 (1.9)	22.1 (1.9)	21.6 (1.9)	–1.21 (90.2)^d^	.23
Age group(years), n (%)				2.09 (2)^e^	.35
18‐20	28 (26.9)	10 (22.7)	18 (30)		
21‐22	37 (35.6)	14 (31.8)	23 (38.3)		
23‐25	39 (37.5)	20 (45.5)	19 (31.7)		
Race/ethnicity, n (%)				1.76 (2)^e^	.41
White	55 (52.9)	20 (45.5)	35 (58.3)		
BIPOC^f^ monoracial	23 (22.1)	10 (22.7)	13 (21.7)		
BIPOC multiracial	25 (24)	13 (29.5)	12 (20)		
Missing	1 (1)	1 (2.3)	0 (0)		
Gender identity, n (%)				1.60 (2)^e^	.46
Nonbinary	69 (66.3)	29 (65.9)	40 (66.7)		
Transgender man	24 (23.1)	12 (27.3)	12 (20)		
Transgender woman	11 (10.6)	3 (6.8)	8 (13.3)		
US Census region, n (%)				7.69 (3)^e^	.05
Northeast	27 (26.0)	7 (15.9)	20 (33.3)		
Midwest	20 (19.2)	6 (13.6)	14 (23.3)		
South	34 (32.7)	19 (43.2)	15 (25)		
West	23 (22.1)	12 (27.3)	11 (18.3)		
Geography, n (%)				4.99 (3)^e^	.14
Urban	27 (26.0)	13 (29.5)	14 (23.3)		
Suburban	64 (61.5)	29 (65.9)	35 (58.3)		
Rural	10 (9.6)	1 (2.3)	9 (15.0)		
Unknown	3 (2.9)	1 (2.3)	2 (3.3)		
Health insurance, n (%)				6.86 (3)^e^	.08
Private	48 (46.1)	17 (38.6)	31 (51.7)		
Public	40 (38.5)	20 (45.5)	20 (33.3)		
Private and public	4 (3.8)	0 (0)	4 (6.7)		
No insurance	11 (10.6)	7 (15.9)	4 (6.7)		
Missing	1 (1)	0 (0)	1 (1.7)		
Own religion, n (%)^g^				1.75 (4)^e^	.81
Christian	7 (6.7)	2 (4.5)	5 (8.3)		
Not Christian	13 (12.5)	7 (15.9)	6 (10)		
Unaffiliated	68 (65.4)	29 (65.9)	39 (65)		
More than one	4 (3.8)	1 (2.3)	3 (5)		
Not applicable	12 (11.5)	5 (11.4)	7 (11.7)		
Family support, n (%)				4.18 (2)^e^	.12
High family support	24 (23.1)	6 (13.6)	18 (30)		
Low family support	31 (9.8)	16 (36.4)	15 (25)		
Not applicable/ They don’t know I’m trans	49 (47.1)	22 (50)	27 (45)		
Family religion, n (%)^h^				7.35 (4)^e^	.11
Christian	55 (52.9)	30 (68.2)	25 (41.7)		
Not Christian	3 (2.9)	1 (2.3)	2 (3.3)		
Unaffiliated	19 (18.3)	6 (13.6)	13 (21.7)		
More than one religion	16 (15.4)	4 (9.1)	12 (20)		
Not applicable	11 (10.6)	3 (6.8)	8 (13.3)		
Mental health therapist, n (%)				1.25 (1)^e^	.26
No	56 (53.8)	27 (61.4)	29 (48.3)		
Yes	48 (46.2)	17 (38.6)	31 (51.7)		
Peer support group, n (%)				0.55 (1)^e^	.46
No	76 (73.1)	30 (68.2)	46 (76.7)		
Yes	28 (26.9)	14 (31.8)	14 (23.3)		
How often sought mental health/ therapy information in adults aged <18 years, n (%)				1.03 (1)^e^	.31
Never or occasionally	45 (43.3)	16 (36.4)	29 (48.3)		
Sometimes, often, or very often	59 (56.7)	28 (53.6)	31 (51.7)		
How useful would it have been to have reliable information on mental health/ therapy in adults aged <18 years, n (%)				0.49 (1)^e^	.49
Not at all or slightly	16 (15.4)	5 (11.4)	11 (18.3)		
Moderately, very, or extremely	88 (84.6)	39 (88.6)	49 (81.7)		
How interested you were in getting therapy related to gender (age <18 years), n (%)				1.78 (2)^e^	.40
Not at all or slightly	43 (41.3)	13 (29.5)	30 (50)		
Moderately, very, or extremely	53 (51)	28 (63.6)	25 (41.7)		
Not applicable	8 (7.7)	3 (6.8)	5 (8.3)		

a PHQ-2: Two-item Patient Health Questionnaire (score ≥3 is the validated cut-off and indicates clinically elevated depressive symptoms).

b Depression was assessed using the PHQ-2.

c Fisher’s exact tests were used to obtain *P* values for cell sizes ≤5.

d *t* test (*df*).

e *χ*2test (*df*).

f BIPOC: Black, Indigenous, and Other People of Color.

g Own Religion: Christian (57.1% Catholic, 14.3% Baptist, 14.3% Orthodox, 14.3% Christian); Not Christian (38.5% Pagan, 30.8% Jewish, 23.1% Buddhist, 7.7% Misotheist); Unaffiliated (41.9% Agnostic, 29.1% Atheist, 19.8% Spiritual, 9.3% Unaffiliated). Percentages may not sum to 100% due to rounding.

h Family Religion (self-reported by TGD young adults): Christian (49.2% Catholic, 20.6% Orthodox, 11.1% Mainline Protestant, 4.8% Evangelist, 3.2% Latter-day Saints, 3.2% Christian, 3.2% Baptist, 1.6% Muslim, 1.6% Jehovah’s Witness, 1.6% Unitarian); Not Christian (66.7% Buddhist, 33.3% Jewish), Unaffiliated (39.3% Atheist, 28.6% Agnostic, 17.9% Spiritual, 14.3% Unaffiliated). Percentages may not sum to 100% due to rounding.

Participants’ mean age was 21.8 (SD 1.9) years; a total of 25/104 (24%) were BIPOC multiracial individuals, while 23/104 (22%) were BIPOC monoracial individuals, and 69/104 (66%) participants identified as nonbinary. The majority (n=64, 61%) of participants resided in a suburban area, and 11/104 (11%) did not have health insurance. Regarding their religious affiliation, 68 (65%) participants described themselves as unaffiliated, while 55 (53%) described their family’s religion as Christian. Half (49/104, 47%) of the participants were not out to their family, 31 (30%) reported low family support, while 24 (23%) participants reported high family support.

Among the participants, 56 (46%) had received mental health therapy and 28 of 104 (27%) had participated in a peer support group. When asked to recall their mental health needs during adolescence: 59 (57%) participants reported seeking information about mental health support or therapy, 88 (85%) indicated that reliable information on mental health support or therapy would have been useful, and 53 (51%) participants expressed having had an interest in therapy related to their gender.

### Correlates of PHQ-2 Depression Score

In bivariate models (Table 2), increased PHQ-2 depression prevalence was associated with (1) being BIPOC multiracial versus White (PR1.39; 95% CI 1.05‐1.84), (2) having low family support versus high (PR 1.53; 95% CI 1.09‐2.15), (3) seeking information about mental health therapy in adolescence (PR 1.31; 95% CI 1.02‐1.67), and (4) being interested in mental health therapy related to gender during adolescence (PR 1.41; 95% CI 1.09‐2.05). Conversely, residing in a rural versus suburban area was associated with decreased PHQ-2 depression prevalence (PR 0.54; 95% CI 0.32‐0.92). No other variables reached statistical significance in bivariate models.

**Table 2.** Bivariate and multivariable Poisson regression models with robust variance estimation: Correlates of Patient Health Questionnaire-2 (PHQ-2)^a^ depression scores in a nationwide sample of transgender, nonbinary, and gender diverse young adults aged 18‐25 across the United States.

Variables	Bivariate models (N=103)^b^, PR^c^ (95% CI)	*P* value	Multivariable model (N=103)^b^, aPR^d^ (95% CI)	*P* value
Age groups (years)				
18‐20	Ref		Ref	
21‐22	0.99 (0.72-1.35)	.94	0.83 (0.55-1.24)	.35
23‐25	1.20 (0.89-1.61)	.24	1.03 (0.69-1.56)	.87
Race/Ethnicity				
White	Ref		Ref	
BIPOC^e^ monoracial	1.10 (0.81-1.50)	.54	0.96 (0.63-1.45)	.84
BIPOC multiracial	1.39 (1.05-1.84)	.03	1.04 (0.75-1.44)	.81
Gender identity				
Nonbinary	Ref		Ref	
Transgender man	1.06 (0.80-1.40)	.70	0.91 (0.64-1.29)	.59
Transgender woman	0.71 (0.45-1.12)	.14	0.69 (0.41-1.16)	.16
US Census region				
Northeast	Ref		Ref	
Midwest	1.19 (0.82-1.73)	.36	1.16 (0.75-1.80)	.51
South	1.31 (0.94-1.80)	.11	1.02 (0.69-1.50)	.94
West	1.31 (0.92-1.87)	.13	1.14 (0.75-1.71)	.54
Geography				
Suburban	Ref		Ref	
Urban	0.96 (0.73-1.27)	.79	0.98 (0.72-1.36)	.93
Rural	0.54 (0.32-0.92)	.02	0.48 (0.26-0.92)	.03
Unknown	1.21 (0.64-2.28)	.57	1.16 (0.58-2.35)	.67
Health insurance				
Private	Ref		Ref	
Public	1.28 (0.99-1.65)	.07	1.08 (0.80-1.46)	.63
Private and public	0.86 (0.42-1.76)	.67	0.91 (0.40-2.05)	.82
No Insurance	1.36 (0.93-1.99)	.11	1.01 (0.63-1.62)	.97
Own religion				
Unaffiliated	Ref		Ref	
Not Christian	1.26 (0.90-1.76)	.19	1.66 (1.04-2.63)	.03
Christian	0.78 (0.45-1.34)	.36	0.76 (0.41-1.41)	.39
More than one religion	0.97 (0.51-1.84)	.93	0.78 (0.38-1.60)	.39
Not applicable	1.07 (0.74-1.55)	.73	1.25 (0.80-1.93)	.32
Family support				
High family support	Ref		Ref	
Low family support	1.53 (1.09-2.15)	.01	1.54 (1.05-2.27)	.03
Not applicable/ they don’t know I’m trans	1.17 (0.85-1.62)	.34	1.06 (0.69-1.60)	.80
Family religion				
Unaffiliated	Ref		Ref	
Not Christian	0.75 (0.32-1.74)	.50	1.61 (0.22-1.68)	.34
Christian	1.09 (0.80-1.49)	.59	1.05 (0.70-1.58)	.80
More than one religion	0.88 (0.58-1.35)	.57	0.86 (0.49-1.51)	.60
Not applicable	0.61 (0.36-1.04)	.07	0.65 (0.33-1.25)	.20
Mental health therapist				
No	Ref		Ref	
Yes	0.92 (0.73-1.17)	.51	0.71 (0.53-0.97)	.03
Peer support group				
No	Ref		Ref	
Yes	1.20 (0.93-1.55)	.16	0.97 (0.68-1.37)	.86
How often sought information on mental health/ therapy by individuals aged<18 years				
Never or occasionally	Ref		Ref	
Sometimes, often, or very often	1.31 (1.02-1.67)	.03	1.15 (0.79-1.66)	.46
How useful would It would have been to have reliable information on mental health/ therapy in individuals aged <18 years				
Not at all or slightly	Ref		Ref	
Moderately, very, or extremely	1.33 (0.92-1.91)	.13	1.18 (0.73-1.92)	.50
How interested you were in getting therapy related to gender in adults aged<18 years				
Not at all or slightly	Ref		Ref	
Moderately, very, or extremely	1.41 (1.09-1.82)	.01	1.47 (0.98-2.21)	.06
Not applicable	1.29 (0.81-2.05)	.29	1.33 (0.80-2.23)	.28

a PHQ-2 score ≥3 indicating clinically elevated depressive symptoms (validated cut-off).

b Models include N=103 participants; 1 participant was excluded due to missing data on health insurance.

c PR: prevalence ratio.

d aPR: adjusted prevalence ratio.

e BIPOC: Black, Indigenous, and Other People of Color.

In a multivariable model (Table 2), after adjusting for all other variables, identifying one’s own religion as not Christian versus unaffiliated (aPR 1.66; 95% CI 1.04‐2.63) and low family support (aPR 1.54; 95% CI 1.05‐2.27) were associated with increased prevalence of positive depression screening. Residing in a rural versus suburban area (aPR 0.48; 95% CI 0.26‐0.92) and ever receiving mental health therapy (aPR 0.71; 95% CI 0.53‐0.97) were associated with decreased PHQ-2 depression prevalence in this model. Age, race or ethnicity, gender identity, US Census region, health insurance, family religion, peer support, history of interest in mental health therapy for gender during adolescence, frequency of information-seeking about mental health resources in adolescence, and desire for reliable mental health information during adolescence, were not significantly correlated with depression based on PHQ-2 scores.

## Discussion

### Principal Results

In this nationwide online study, depression identified using PHQ-2 scores was highly prevalent, with more than 4 in 10 TDG young adults showing clinically elevated symptoms. Low levels of family support and nonChristian religious affiliation were associated with an increased likelihood of positive PHQ-2 depression screening. This exploratory study highlights the high rates of depressive symptoms among TGD young adults as well as the need for tailored interventions to improve mental health among those at the highest risk.

### Comparison With Prior Work

This study found that TGD young people with low family support had a higher prevalence of PHQ-2 depression compared to those with highly supportive families. This finding corroborates prior research on the protective role of family support on TGD young peoples’ mental health and has informed intervention development for the broader TransHealthGUIDE study [15,16]. For example, a key intervention component that has been developed for the overall study includes app-based education for TGD young adults and their caregivers on ways to improve family communication, connection, and support.

No differences in PHQ-2 depression prevalence were found for family religion, despite religious affiliation influencing attitudes toward TGD issues in the United States [18,19]. The TGD young people identifying their own religion as nonChristian (eg, Jewish, Muslim, Buddhist) had a higher prevalence of PHQ-2 depression than those identifying as unaffiliated; however, no significant differences were found between Christian and unaffiliated TGD young adults. The association between religious affiliation and depression has been inconsistent in prior research on young people [25], including a recent systematic review of religion, spirituality, and mental health among TGD youth [17]. Future mental health research may benefit from measuring not only religious affiliation but also the degree of religiosity, which was not assessed in this study.

This study found that a history of mental health help-seeking, specifically in young adults receiving mental health therapy was significantly associated with reduced PHQ-2 depression prevalence. This highlights the need for interventions that connect TGD young adults to necessary mental health services. The American Academy of Pediatrics recommends that adolescents aged 12 years and older be screened annually for depression using a formal self-report screening tool to identify and treat those with depression early in the course of illness, potentially reducing long-term depression-related morbidities [26]. This finding underscores the importance of improving mental health care access for TGD young adults to address depression, including telehealth and other online or digitally delivered services and interventions [27].

Regarding sociodemographic differences, we found no evidence of gender identity differences in PHQ-2 depression prevalence, inconsistent with prior research demonstrating the highest depression burden among nonbinary young adults [9]. However, previous studies have reported mixed findings regarding gender identity differences in depression [13], suggesting the need for ongoing research. While BIPOC multiracial TGD young people exhibited elevated PHQ-2 depression in bivariate analyses, consistent with national US population data, this finding did not remain significant in the adjusted model [3]. Geographic context emerged as a significant factor in this study, in alignment with psychiatric epidemiologic research showing lower depression rates in rural settings in high-income countries [28]. The TGD young adults residing in rural areas had lower PHQ-2 depression prevalence compared to those living in suburban areas. Prior studies have found greater mental distress symptoms among rural versus nonrural TGD adults but did not distinguish between the urban and suburban subgroups [29]. More recent research identified the lowest levels of depressive symptoms in rural versus urban-dwelling TGD adults [30]. Additional research is needed to better understand PHQ-2 depression prevalence and set of symptoms among TGD young adults in rural, urban, and suburban settings.

### Limitations

These findings should be interpreted alongside this study’s limitations. First, analyses were exploratory, and the small sample size limits statistical power to detect significant associations; the findings will require replication. Further, given the cross-sectional design, the study findings are associational only, and longitudinal research is recommended. Second, related to study measures, the global measure of family support may have overlooked important details about family dynamics, such as communication, acceptance, and explicit expressions of care and support [31]. Relatedly, mental health care utilization variables were self-reported rather than objectively measured. Future research that includes self-reported data alongside electronic health record data would strengthen the evidence base. Third, this study did not assess nonpsychiatric aspects of participants’ medical histories, and unmeasured confounding due to gender-affirming medical treatments cannot be ruled out [10,12]. Lastly, there are few validated measures for assessing mental health help-seeking behaviors [32]. Further research to understand online help-seeking behaviors for TGD young people using validated measures is warranted to inform future interventions [33].

### Conclusions

The high prevalence of PHQ-2 depression in this online sample of TGD young adults underscores the importance of universal screening for depression in this population. It also highlights the need for appropriate systems to ensure prompt diagnosis, treatment, and follow-up for TGD young adults with PHQ-2 depression. Further, tailored interventions may be necessary for subgroups at increased risk for depression, such as TGD young adults with low family support, to decrease mental health disparities. The continued expansion of online interventions may facilitate the engagement of TGD young adults who would benefit from mental health prevention and treatment efforts [27]. This study’s findings have important implications for clinical care and the design, development, and implementation of interventions aimed at improving mental health outcomes and reducing health disparities among TGD young adults.

## Abbreviations

aPR: adjusted prevalence ratio
BIPOC: Black, Indigenous, and People of Color
PHQ-2: two-item patient health questionnaire
PR: prevalence ratio
TGD: Transgender, nonbinary, gender diverse
